# The Utility of Color Doppler to Confirm Endotracheal Tube Placement: A Pilot Study

**DOI:** 10.5811/westjem.2020.5.45588

**Published:** 2020-07-10

**Authors:** Thomas H. Gildea, Kenton L. Anderson, Kian R. Niknam, Laleh Gharahbaghian, Sarah R. Williams, Timothy Angelotti, Paul S. Auerbach, Viveta Lobo

**Affiliations:** * Stanford University School of Medicine, Department of Emergency Medicine, Palo Alto, California; † Stanford University School of Medicine, Department of Anesthesiology, Perioperative and Pain Medicine, Palo Alto, California

## Abstract

**Introduction:**

Grayscale ultrasound (US) imaging has been used as an adjunct for confirming endotracheal tube (ETT) placement in recent years. The addition of color Doppler imaging (CDI) has been proposed to improve identification but has not been well studied. The aim of this study was to assess whether CDI improves correct localization of ETT placement.

**Methods:**

A convenience sample of emergency and critical care physicians at various levels of training and experience participated in an online assessment. Participants viewed US video clips of patients, which included either tracheal or esophageal intubations captured in grayscale or with CDI; there were five videos of each for a total of 20 videos. Participants were asked to watch each clip and then assess the location of the ETT.

**Results:**

Thirty-eight subjects participated in the online assessment. Levels of training included medical students (13%), emergency medicine (EM) residents (50%), EM attendings (32%), and critical care attendings (5%). The odds ratio of properly assessing tracheal placement using color relative to a grayscale imaging technique was 1.5 (p = 0.21). Regarding the correct assessment of esophageal placement, CDI had 1.4 times the odds of being correctly assessed relative to grayscale (p = 0.26). The relationship between training level and correct assessments was not significant for either tracheal or esophageal placements.

**Conclusion:**

In this pilot study we found no significant improvement in correct identification of ETT placement using color Doppler compared to grayscale ultrasound; however, there was a trend toward improvement that might be better elucidated in a larger study.

## INTRODUCTION

Determination of correct endotracheal tube (ETT) placement is an essential part of airway management. Unrecognized esophageal intubation can lead to morbidity and mortality due to hypoxia and iatrogenic aspiration due to stomach insufflation. There are multiple methods to confirm correct ETT tube placement that include direct visualization, capnography, visualization of chest rise, and direct auscultation; however, each of these methods has its limitations.^1^,^2^ Quantitative waveform capnography combined with clinical assessment comprises the most reliable method to confirm ETT placement. However, the utility of capnography can be limited in cases of poor cardiac output, low pulmonary blood flow, airway obstruction, or after the administration of epinephrine.^2^–^4^

Grayscale ultrasound (US) has become an increasingly popular adjunct for confirming ETT placement in recent years. US is widely available in emergency department settings, and there is a growing base of evidence supporting its use to confirm ETT placement^5^–^9^; however, even this technique can be limited by patient anatomy or user experience. The addition of color Doppler imaging (CDI) has been proposed to improve ETT localization by highlighting ETT movement, but this technique has not been well studied.^10^,^11^ Only one prior investigation has compared the use of CDI with grayscale US for confirming ETT placement, and that study did not find a difference between the techniques in a cadaver model.^11^ However, cadaver tissue often appears different from and lacks the respiratory variations of live human tissue with ultrasound imaging; thus, a cadaver model may not accurately reflect the performance of CDI in confirming ETT placement in live humans.^12^,^13^

In this pilot study, we used video captured from live humans, with either tracheal or esophageal intubations, to evaluate whether CDI can improve correct ETT localization compared to grayscale US using an online assessment of medical professionals. We also investigated whether there is a relationship between the accuracy of US interpretation for this indication and the training level of participants.

## METHODS

### Experimental Design and Participants

This was a convenience sample of emergency and critical care attending physicians, resident physicians, and medical students from a single academic hospital who participated in an online assessment. Subjects were recruited via email with a link to the online assessment. This study was approved by the institutional review board (IRB); subjects provided informed consent.

### Video Clips

The online assessment used looped, six-second, trans-tracheal ultrasound video clips from a de-identified archive of patients who were intubated in the operating room prior to elective surgery. The archive was collected over a two-year period as part of a separate IRB-approved research study, and the video clips were maintained for educational and research purposes. Anesthesiologists performed all the intubations, and emergency ultrasound fellowship-trained physicians performed all the ultrasound examinations. The video clips were obtained using Sonosite Edge I ultrasound machines (Sonosite, Bothell, WA) equipped with a high-frequency linear transducer (L25x) that was applied at the level of the cricothyroid membrane. The video archive included video images of both tracheal and esophageal intubations. Intentional esophageal intubation was briefly performed on a subset of patients prior to endotracheal intubation; the location of each intubation was verified by direct visualization, auscultation of the stomach and lungs, and quantitative capnography. The location (esophageal vs tracheal) of each intubation was noted and kept in the archive, but no labels were present on the clips used in the online quiz. All clips were recorded post-intubation, using grayscale and/or CDI. CDI clips were captured while longitudinally oscillating the ETT manually.

**Population Health Research Capsule**

What do we already know about this issue?

*Grayscale ultrasound is an adjunct for confirming endotracheal tube placement. The addition of color Doppler imaging (CDI) has been proposed to improve localization*.

What was the research question?

Does CDI improve correct endotracheal tube localization compared to grayscale ultrasound?

What was the major finding of the study?

*In this pilot study, there was no significant improvement in localization using CDI*.

How does this improve population health?

*This pilot study suggests that CDI may not provide much clinical value compared to grayscale imaging alone*.

**Figure 1:**
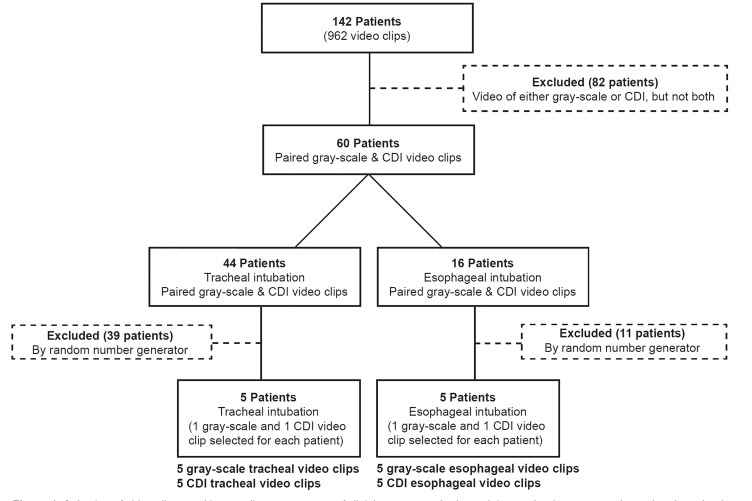
Selection of video clips used in an online assessment of clinician accuracy in determining tracheal versus esophageal endotracheal tube placement using color Doppler imaging (CDI) relative to grayscale imaging technique.

The entire video archive contained a total of 964 clips from 142 patients (Figure 1). Each patient in the archive had a range of 3–14 clips of either a tracheal or an esophageal intubation; 82 of the subjects had clips captured in either grayscale or CDI, and 60 of the patients had pairings acquired in both grayscale and CDI. For this study, video clips were selected from 10 of the 60 patients, which had pairings of the same ETT placement (tracheal or esophageal) acquired in both grayscale and CDI. A total of 44 patients with tracheal intubations and 16 patients with esophageal intubations met this criterion. Five patients from each of these two groups were selected using a random number generator. One grayscale and one CDI video were used from each of these 10 patients to yield a total of 20 video clips that were included in the online assessment (Figure 1). If patients had more than the two required video clips in the archive, the video with the earliest time stamp was used unless the color Doppler box had been placed incorrectly. To reduce bias we excluded videos that were located to one side or the other of the screen rather than centered over both the trachea and esophagus.

### Online Assessment

**Figure 2:**
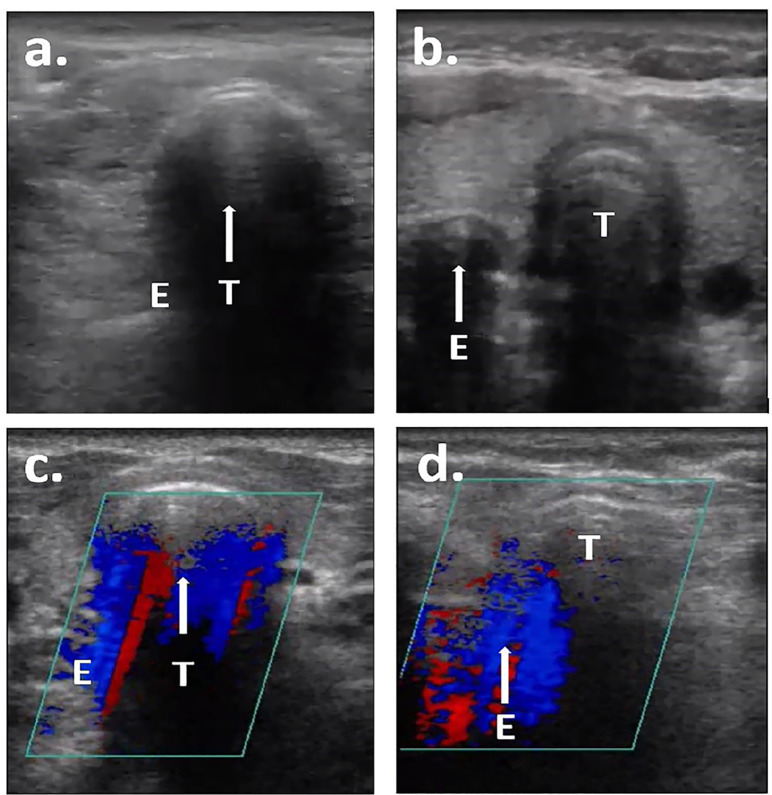
Representative screenshots from online assessment videos: a) tracheal intubation in grayscale (single air-mucosal interface with reverberation artifact within the trachea); b) esophageal intubation in grayscale (double air-mucosal interface); c) tracheal intubation in color Doppler imaging (CDI) (color signal deep to the trachea); and d) esophageal intubation in CDI (color signal deep to the esophagus). Arrow indicates location of intubation. *T*, trachea; *E*, esophagus.

The online assessment included a seven-minute instructional video (Supplement 1), a background survey, and an ETT placement quiz. The instructional video demonstrated the proper interpretation of grayscale and CDI transtracheal images (Figure 2). The clips in the instructional video were not used in the online quiz. The background survey queried participants about their medical specialty, level of training, and whether they had any specialized training in ultrasound (“fellowship trained or ultrasound faculty”). The assessment consisted of 20 questions; each question was comprised of a single video clip that participants were asked to identify as either an esophageal or tracheal intubation. The assessment included an equal number of esophageal and tracheal intubations, and it included both the grayscale and CDI clips from the pairs described above. The assessment was administered using Qualtrics (Qualtrics, Provo, UT). To establish face validity of the online assessment and to ensure the quality of the content, four independent emergency ultrasound fellowship-trained physicians were included in a trial run of the assessment prior to enrollment of study participants.

### Statistical Analysis

Continuous data are presented as means with standard deviations (SD). Odds ratios (OR) are reported for categorical frequency data. We used a mixed-effects logistic regression model to adjust for clustering when determining the effects of properly assessing grayscale vs color and to observe the relationship between correct assessments and training level.

## RESULTS

A total of 38 subjects participated in the online assessment. The training level for participants and corresponding mean quiz scores are shown in Table 1. Three of the EM attending physicians (8% of the total subjects, 25% of the EM attendings) had specialized training in ultrasound; no other participant had specialized ultrasound training. The mean quiz score for the three attendings with specialized ultrasound training was 19 (SD 1.0; 95% confidence interval).^14^^–20^ The OR of properly assessing tracheal or esophageal placement with CDI relative to a grayscale imaging technique is shown in Table 2. The relationship between training level and correct assessments was not statistically significant in either the tracheal or esophageal placements.

## DISCUSSION

This is the first study describing the potential utility of CDI to confirm ETT placement via transtracheal ultrasound in live human subjects. In this pilot study we found no significant improvement in correctly identifying tracheal or esophageal placement with CDI relative to grayscale imaging. However, there was a trend toward improved correct identification with CDI, which merits further study. Further prospective work should be performed to determine whether CDI adds a benefit when used in real time.

In our study, participants generally identified the correct ETT placement location approximately 85% of the time with both grayscale imaging and CDI. These results are lower than the findings of three recent systematic review and meta-analyses that described sensitivities and specificities of >93% and >97%, respectively.^14^–^16^ There are several factors that might contribute to this difference. First, most of the studies included in the meta-analyses had operators interpreting images in real time; this real-time control over the imaging and the tactile input could help participants correctly interpret images. Second, in the archive from which our videos for this study were drawn, not all images were of equal quality, and videos were selected randomly to decrease bias. It is possible that some lower quality videos could have negatively impacted our results, although all the videos were reviewed by point-of-care ultrasound experts prior to enrollment, and none were deemed to be technically limited. When images are being interpreted in real time, at the point of care, if the operator is dissatisfied with a view, he or she can adjust until a satisfactory image is acquired. Additionally, most of the participants in this study had no prior experience with transtracheal ultrasonography and relied on a seven-minute video for training. In contrast, most of the studies included in the meta-analysis put their participants through hours of training, which may be another explanation for the difference in results. Lower accuracy has been described in other recent studies in which participants had less training time. For example, Gottlieb et al trained residents on transtracheal ultrasound in 10 minutes and reported a sensitivity and specificity of 95.5% and 71.7%, respectively.^17^ Additionally, Stuntz et al trained participants by distributing a paper handout to participants a week before and again on the day of the study; this investigation reported a sensitivity and specificity of 62.0% and 37.9%, respectively.^18^ In our group, the physicians with specialized ultrasound training had quiz scores that trended higher than the other groups, which also suggests that additional ultrasound training may improve accuracy; however, due to the variance attributable to small sample size, a larger study would be required to verify this supposition.

Although we were unable to demonstrate a significant difference between assessing tracheal or esophageal placement with CDI relative to a grayscale imaging technique, we did see a trend toward improvement with CDI in correctly identifying both. This was especially interesting given the fact that this trend was seen over a wide range of interpreter experience levels. A larger study may verify this implication, yet the question of clinical significance remains. A post-hoc sample-size calculation revealed that a comparison of more than 50 subjects would be necessary to detect a difference between CDI and grayscale performance, which raises the question about the circumstances under which CDI might be clinically useful. The need for a larger sample size suggests that CDI may be useful occasionally, but perhaps not very frequently. Nonetheless, the addition of CDI only takes a few extra seconds to perform and may have some benefit in certain cases.

Another unanswered question is whether power Doppler may improve assessment compared to either CDI or grayscale imaging. Power Doppler is a newer ultrasound technique that has a greater sensitivity to detect movement compared to conventional CDI; thus, it may be able to provide more information about the subtle movements of an ETT.

## LIMITATIONS

Participants in this study represent a convenience sample that was recruited via email; this is a potential source of selection bias. Participants who were more confident in their ultrasound abilities may have enrolled at a higher rate, which would blunt the difference between the physicians with specialized training and those without. Additionally, we did not assess retention of knowledge in this study. More work on the retention of point-of-care ultrasound skills and knowledge is needed in general; this is not limited to the use of CDI for airway management. Finally, although airway ultrasound may be useful in a variety of settings there was not an equal number of critical care and emergency physicians included in this study. Nonetheless, this pilot study provides a quantitative reference for the difference between grayscale and CDI among physicians with varied ultrasound experience that can be used to conduct larger prospective investigation on the topic.

## CONCLUSION

In this pilot study we found no significant improvement in correct identification of ETT placement using color Doppler imaging compared to grayscale imaging; however, there was a trend toward improvement, over a wide range of interpreter experience levels, that might be better elucidated in a larger study.

## Supplementary Information

Video Excerpt from the instructional video demonstrating how to recognize an esophageal intubation with Color Doppler Imaging.

## Figures and Tables

**Table 1:** Participant specialty and training levels.

Training level	N (%)	Mean Quiz Score [SD]	95% CI
Medical student	5 (13)	17 [1.9]	14–19
Emergency medicine resident	19 (50)	17 [1.6]	17–18
Emergency medicine attending	12 (32)	17 [1.7]	16–19
Critical care attending	2 (5)	14 [0.71]	7.1–20

*SD*, standard deviation; *CI*, confidence interval.

**Table 2:** Results of online quiz testing how accurately clinicians confirm ETT* placement when using grayscale vs color Doppler imaging techniques (CDI).

ETT Location	Grayscale Questions Correct, Total (%)	CDI Questions Correct, Total (%)	Odds Ratio, (95% CI)	p-value
Trachea	162 (85%)	170 (89%)	1.5 (0.8–2.7)	0.21
Esophagus	155 (82%)	163 (86%)	1.4 (0.8–2.4)	0.26

*ETT*, endotracheal tube; *CI*, confidence interval.
